# Improving equity in prehabilitation before cancer surgery: consensus‐based considerations for leaders and practitioners: a nominal group technique study*

**DOI:** 10.1002/anr3.70085

**Published:** 2026-07-14

**Authors:** X. Zhang, L. Ashmore, C. Hadley, C. Kullikowski, S. Stanley, H. Stewart, L. Wareing, C. Gaffney, A. Partridge, A. Smith, J. Rycroft Malone, C. Shelton

**Affiliations:** ^1^ Lancaster Medical School Lancaster University Lancaster UK; ^2^ North West School of Anaesthesia, NHS England Workforce Training and Education North West Manchester UK; ^3^ Patient Co‐Investigator Lancaster UK; ^4^ Department of Anaesthesia, Royal Lancaster Infirmary University Hospitals of Morecambe Bay NHS Foundation Trust Lancaster UK; ^5^ Faculty of Health and Medicine Lancaster University Lancaster UK; ^6^ Department of Anaesthesia, Wythenshawe Hospital Manchester University NHS Foundation Trust Manchester UK

**Keywords:** consensus workshop, equity, good practice considerations, nominal group technique, prehabilitation

## Abstract

Prehabilitation aims to improve physical, nutritional and psychological health before surgery. Despite its potential benefits, inequitable access remains a concern. The wider PARITY study aimed to identify and help address inequalities in prehabilitation before cancer surgery using a mixed‐methods approach. This report describes the end‐of‐study prioritisation workshop, which brought together professional, public and patient participants to prioritise considerations to promote equity in prehabilitation services using a nominal group technique. Before the workshop, 42 considerations identified from preceding work, including co‐design, Delphi, service mapping and case studies, were shared with participants for review. Participants (n = 15) discussed and prioritised these considerations according to perceived impact and feasibility of implementation. Considerations were ranked by perceived priority and categorised by level of implementation. Forty‐two considerations were prioritised and categorised as system level (n = 15), service level (n = 22) and practitioner level (n = 5). The six highest‐priority considerations relate to: understanding how prehabilitation and surgery may affect each patient's life; adapting delivery to individual needs; making services available at a system‐wide level; contacting patients who are not engaging; providing interpreters where needed; and training care teams in equality and diversity issues. These findings provide consensus‐based considerations to support more equitable prehabilitation before cancer surgery, at a time when prehabilitation is increasingly prominent in national guidance.

## Introduction

Prehabilitation can enhance patients' functional capacity before major surgical treatment, thereby improving health outcomes and enhancing recovery [1]. Its core components comprise physical exercise, nutritional optimisation and psychological support. By reconceptualising waiting time before an operation as ‘preparation time’, prehabilitation seeks to create the conditions to improve fitness and functional ability [2, 3].

Access to prehabilitation services in the UK is inequitable at present [4], which has the potential to undermine the potential benefits for the most disadvantaged groups. Service delivery varies across regions, organisations and healthcare settings, resulting in unequal engagement within and between populations. Social determinants of health, such as socioeconomic status, ethnicity and geographic location, can influence access and engagement [5, 6].

Prehabilitation is a complex intervention requiring sustained engagement. This has the potential to be resource‐intensive for both patients and services [6]. Patients may face challenges, such as physical symptoms, financial burden, transport difficulties and competing personal priorities. Service providers may struggle with limited staffing and funding, variable service models and limitations in facilities [6, 7, 8]. This makes equitable delivery particularly challenging.

Recent national guidance has emphasised the importance of addressing inequalities. The Macmillan Cancer Support ‘Prehabilitation for Cancer Clinical and Implementation Guidance 2025’ recommends that health inequalities should be explicitly considered in the design, delivery and implementation of prehabilitation, to avoid exclusion and ensure equitable access [9]. However, there remains limited evidence on how to operationalise equitable prehabilitation in practice.

The PARITY (Prehabilitation for Cancer Surgery: Quality and Inequality) study was designed to address this gap by understanding inequalities in access to prehabilitation before cancer surgery and identifying strategies to address them. It included three sub‐studies: a co‐design and Delphi‐based study involving patients, carers and professionals that explored the aims, objectives and values of prehabilitation [10]; a national mapping study of prehabilitation available to patients awaiting cancer surgery in UK National Health Service (NHS) hospitals, including how inequalities were addressed within services [4]; and qualitative case studies of six diverse NHS services to explore patient experiences, service delivery models and strategies used to improve accessibility and inclusivity [11].

Across the three sub‐studies [4, 10, 11], we drew on multiple methods to identify 42 potential ways to promote equitable engagement with prehabilitation. However, our sub‐studies were not designed to identify which approaches were most effective or determine whether approaches implemented in one service could be used in another, and the wider evidence base for prehabilitation remains incomplete [6].

Consensus processes are used to generate recommendations based on experience where evidence is uncertain and multiple perspectives should be represented [12]. The final stage of the PARITY study was to draw the insights from the prior stages of the study together and consider their potential for impact and implementation. A consensus workshop was conducted with patient and public representatives, health professionals and health service leaders; participants were asked to draw on their knowledge and experience to prioritise considerations based on their perceived impact and feasibility, with the aim of informing a best practice guide for equitable prehabilitation services for patients awaiting cancer surgery in UK NHS services.

## Methods

Reporting of this study is informed by the ACcurate COnsensus Reporting Document (ACCORD) guidelines [12]. We conducted a structured, consensus‐based priotisation workshop to rank the 42 considerations identified in prior elements of the study. The wording of these was agreed by the PARITY study team following review of findings from the other phases of the project. The workshop employed a nominal group technique, a structured consensus method which facilitates small group discussion, individual reflection and iterative consensus‐building [13]. It enables prioritisation of ideas through rounds of individual ranking, small group discussion and full panel review. This allows participants to reflect independently and collectively while ensuring that all voices are heard. It supports transparent consensus‐building by integrating quantitative rankings with qualitative discussion. The workshop took place on a single day in July 2025, with options to attend in‐person (Manchester, UK) or online. Travel arrangements and expenses were covered where needed, and public participants were reimbursed in line with National Institute for Health and Care Research (NIHR) guidance.

Multidisciplinary stakeholders were identified by the PARITY research team based on their role in prehabilitation (professional or lived‐experience expertise) through relevant professional groups and via involvement in earlier stages of the study. Invitations were sent via email. A summary of the 42 considerations for equitable prehabilitation before cancer surgery (Supporting Information Appendix S1) was sent to participants via email 2 weeks before the workshop to allow sufficient time for review and familiarisation. We aimed to recruit between 10 and 15 participants to have enough for two small groups, consistent with previous work suggesting an optimal small‐group size of 5–10 participants [13, 14].

The workshop was conducted according to a facilitation guide (Supporting Information Appendix S2). It commenced with a plenary session in which the summary list was recapped. Participants were then divided into two groups, ensuring a mix of expertise between prehabilitation providers, health service leaders and patient and public participants within each group (group 1, n = 8; group 2, n = 7). Within each group, participants were asked to identify the three recommendations they considered most important (‘top three’) and least important (‘bottom three’) independently, based on impact and feasibility. This was followed by parallel facilitated group discussions.

**Figure 1:**
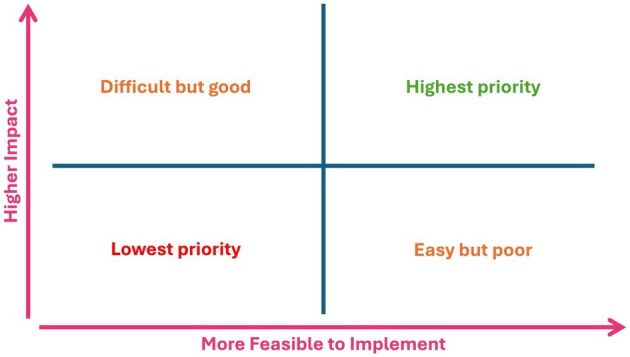
Impact–feasibility matrix used as a visual aid for the workshop.

The first session commenced with each participant articulating their ‘top three’ and ‘bottom three’ considerations and then working together to agree a ranked order. An online collaboration platform (Miro, RealtimeBoard Inc, Amsterdam, Netherlands) was used to display the ranked order as it was developed. An impact–feasibility matrix (Figure 1) was used as a visual aid to assist the participants to prioritise the recommendations, with the highest‐priority recommendations being both impactful and feasible.

In the afternoon, participants were reassigned into new small groups to promote exposure to different viewpoints and optimise group dynamics. Each group was provided with a preliminary ranked list derived from the average rankings of the morning groups. Participants reviewed and discussed this list and worked collaboratively to agree on a revised prioritised order.

The final part of the workshop was a facilitated plenary session where all participants reviewed and discussed the aggregated ranking generated from the afternoon outputs and agreed on the final prioritisation.

Rankings from group exercises were aggregated by calculating average positional scores for each recommendation. These scores were used to generate preliminary ranked lists at each stage of the workshop.

The final prioritised list was reviewed during the concluding plenary discussion, with the opportunity to re‐order items with the agreement from the group. This ensured that the final recommendations reflected both aggregated quantitative input and group agreement. Participants were briefed that any decisions in this session should be by unanimous consent, although a majority vote could be used, if agreement was not possible.

Ethical approval was granted by Lancaster University Faculty of Health and Medicine Research Ethics Committee in June 2025 (reference: FHM‐2022‐1063‐RECR‐1).

## Results

The workshop panel included 15 participants representing a range of perspectives, including patient and public voice (n = 5) and professionals involved in prehabilitation (n = 10). Twelve participants identified as female and three as male; two participants (both male) identified as belonging to a minority ethnic group.

The small group sessions proceeded as specified in the protocol, except for one participant needing to leave to attend another commitment before the conclusion of the afternoon session. Facilitators noted that discussions became passionate on occasion, and that some participants were more dominant than others. This was addressed through the structure of the approach (with everyone guaranteed the opportunity to speak) and active management of group dynamics [13]. In the concluding plenary discussion, the group discussed and agreed the ordering of three considerations which had the same average rank (Supporting Information Appendix S3), and the remaining participants unanimously agreed the order of considerations.

The agreed list was subsequently shared with the entire group, who suggested some minor changes to the wording of some items for consistency of style; the final prioritised list was confirmed by unanimous consent. The six highest‐priority considerations are outlined in Box 1.

The full list of considerations, classified by their primary level of implementation, is presented in Table 1. Interim and final rankings are presented in Supporting Information Appendix S3.

Box 1 **Highest‐priority considerations for equitable prehabilitation before cancer surgery.**

1. There should be a conversation to understand how a patient's prehabilitation care plan and subsequent cancer surgery will affect their life.
2. Prehabilitation services should be prepared to adapt the way they are delivered to suit the needs of individual patients and facilitate their participation.
3. Prehabilitation services should be made available on a ‘system‐wide’ level (across the geographical footprint of a healthcare organisation)
4. Prehabilitation services should include outreach to contact patients who do not appear to be engaging and identify barriers to participation.
5. Interpreters should be provided for those who need them, including for sensory impairment, at each appointment.
6. The care team should be trained to improve their understanding of equality and diversity issues.

**Table 1:** Full list of considerations, classified by primary level of implementation. Within each implementation level, lower numbers indicate higher priority.

**System‐level considerations (for strategic leaders)**	
1	There should be a conversation to understand how a patient's prehabilitation care plan and subsequent cancer surgery will affect their life.
2	Prehabilitation services should be made available on a ‘system‐wide’ level (across the geographical footprint of a healthcare organisation).
3	Where a prehabilitation service covers a large geographical area, activities should be spread across that area (using local healthcare facilities/community leisure centres) to minimise patient travel distances.
4	Professional communication related to prehabilitation should be shared/integrated with other professionals involved in the patient's care (primary care, surgical team).
5	Prehabilitation services should negotiate discounted access to leisure facilities, to facilitate patients' participation in exercise.
6	Prehabilitation services should loan equipment (exercise equipment, digital equipment) to patients to facilitate their engagement in prehabilitation.
7	Prehabilitation activities should be made available using digital technologies (video‐conferencing), to facilitate participation without the need for in‐person attendance.
8	Prehabilitation activities should be made available on a face‐to‐face basis, to facilitate participation without access to digital/telephone facilities.
9	Prehabilitation services should reimburse/provide travel expenses to facilitate the attendance of patients who are less able to afford travel.
10	Patients with learning disabilities should be enabled to participate in prehabilitation, in collaboration with learning disability nurses/teams.
11	Prehabilitation activities should be made available using telephone, to facilitate participation without the need for in‐person attendance.
12	Prehabilitation services should provide food vouchers to support patients who are less able to afford food to engage with dietary recommendations.
13	Prehabilitation services should provide travel/transport services, to enable attendance by patients who are less able to travel independently.
14	Where patients are unable or less able to travel to attend prehabilitation services, home visits should be offered.
15	Prehabilitation services should arrange local accommodation to facilitate the participation of patients from out of area.
**Service‐level considerations (for prehabilitation service leads)**	
1	Prehabilitation services should be prepared to adapt the way they are delivered to suit the needs of individual patients and facilitate their participation.
2	Prehabilitation services should include outreach to contact patients who do not appear to be engaging and identify barriers to participation.
3	Interpreters should be provided for those who need them, including for sensory impairment, at each appointment.
4	The care team should be trained to improve their understanding of equality and diversity issues.
5	Prehabilitation services should provide a wide range of appointment times, to enable participation by patients who have time‐bound commitments (work, caring responsibilities).
6	Prehabilitation staff who provide dietary advice should be trained on cultural and religious dietary conventions and requirements.
7	When patients are expected to undertake administration to participate in prehabilitation (booking appointments, arranging transport), they should be offered support to complete this.
8	Prehabilitation should take place in facilities which are fully accessible to patients with restricted mobility.
9	Where digital data entry is needed (during screening), patients should be offered support to complete this.
10	Digital resources (videos, images) should include subtitles/metadata.
11	Prehabilitation teams should make referrals to support services (charities, social services, social prescribers) where a need is identified.
12	Prehabilitation teams should signpost community resources (leisure centres) which have gender‐specific facilities (women‐only gyms).
13	Prehabilitation literature (patient information leaflets) should be made available in paper form, to facilitate engagement by patients without access to digital facilities.
14	Prehabilitation teams should signpost patients to financial support services (charities, credit unions).
15	Prehabilitation services should refer patients who are from out of area to local prehabilitation programmes to facilitate their participation.
16	Prehabilitation services should facilitate peer support between patients.
17	Prehabilitation literature (information leaflets) should be available in languages which are commonly used by the local population.
18	Appointments to attend prehabilitation activities should be available to suit diverse religious/cultural calendars.
19	Prehabilitation services should have a clear and dedicated means of contact for patients (telephone hotline).
20	Prehabilitation services should have a group of ‘key contacts’ to provide advice for caring for patients with protected characteristics and vulnerable people when they are referred.
21	Prehabilitation teams should accommodate patients who wish to be cared for by people of their own gender.
22	Prehabilitation teams should signpost retailers which supply exercise clothing appropriate for patients' religious/cultural preferences.
**Practitioner‐level considerations (for prehabilitation practitioners)**	
1	Prehabilitation teams should acknowledge and normalise that patients may find the activities involved in prehabilitation challenging (due to physical fitness and/or symptoms).
2	Patients' relatives and carers should be included in prehabilitation appointments, if the patient expresses a preference for this to take place.
3	Prehabilitation teams should encourage patients to engage in physical activity based on their usual everyday activities (walking the dog).
4	Prehabilitation activities should be adapted to facilitate the participation of people living with disabilities.
5	Prehabilitation staff who provide dietary advice should accommodate patients' preferences when developing nutrition plans.

The findings of the workshop were summarised in a plain English document, which is included in Supporting Information Appendix S4.

## Discussion

This element of the PARITY study prioritised considerations for equitable delivery in UK prehabilitation services, based on impact and feasibility of implementation. This supports the growing recognition that the value of prehabilitation depends not only on intervention content, but also on how services are designed, communicated and delivered within healthcare systems [6, 9, 11].

The highest‐priority consideration identified was the need for explicit conversations with patients to enable them to understand how prehabilitation and the subsequent cancer surgery may affect their lives. While information sharing and consent are central to all healthcare interventions, in the context of prehabilitation, discussions such as these generate opportunities to identify individual needs, preferences and potential barriers, enabling care plans to be tailored individually. This finding is consistent with studies which emphasise the importance of shared decision‐making and patient‐centred communication [15]. Previous studies have also reported that personalised approaches to prehabilitation result in greater improvements than standardised delivery [16, 17].

Participants identified that prehabilitation services should adapt to suit individual patient needs. Flexible delivery models, including face‐to‐face, remote and hybrid approaches, may help accommodate work schedules, mobility limitations, caregiving responsibilities or other competing demands. A needs‐based, person‐centred approach could help patients who might otherwise be unable or unwilling to participate in prehabilitation, for example due to personal reasons or logistical constraints. In addition, practical support, such as local delivery, transport assistance and provision of exercise equipment or internet access, was recognised as an important factor. Addressing these barriers may improve programme engagement, with potential downstream benefits for clinical outcomes.

Participants highlighted the importance of providing interpreters and developing easily accessible services. Because prehabilitation requires active engagement and often involves behavioural change [8, 9], addressing communication barriers could improve patients' understanding and participation and increase the accessibility and equity of services. The availability of interpreters and accessible communication is also important to ensure informed consent and equitable access. These findings are consistent with broader research, highlighting the influence of social and structural determinants on engagement and outcomes [6, 7, 18].

Participants identified that system‐wide delivery across all healthcare organisations within a region would contribute to equitable access. Fragmented or localised service provision may result in geographical disparities [11], limiting access for some populations, especially those from deprived or remote areas who are more likely to experience health inequalities related to geography. Evidence suggests that integrated prehabilitation delivered at a system level may improve referral consistency, programme outreach, continuity of care and cost‐effectiveness [19, 20].

Training healthcare staff to improve their understanding of equality and diversity issues was identified as a priority for prehabilitation services. Healthcare professionals equipped with cultural competence are better able to provide accessible, responsive and respectful care to diverse patient populations, especially for patients from minority or underserved groups who may face additional barriers. Previous research has indicated that patients from some minority ethnic groups are less likely to engage with prehabilitation [6], and improving staff knowledge and awareness of diversity and equality may enhance patient‐healthcare professional relationships and foster mutual understanding, which may support more person‐centred and culturally appropriate care, with implications for patient satisfaction, adherence and health outcomes [21].

While the feasibility of implementation was explicitly weighed in the prioritisation process, we acknowledge that UK health services are operating in a resource‐constrained environment at present. This means that there are some underlying resource requirements that will need to be put in place to ensure that these approaches are sustainable in the long term and do not place undue strain on prehabilitation teams.

Limitations of this study include that the panel was small and, compared to both the UK population and the UK health workforce [22], female participants (12/15) were relatively over‐represented and people from minority ethnic groups (2/15) were relatively under‐represented. This may mean that some viewpoints were given greater prominence than in the general population. Professionals (10/15) represented a greater proportion of participants than patient and public participants (5/15), and this, combined with the possibility that professionals may be more confident when engaging in group discussions, may have influenced the outcome, despite the facilitators' focus on ensuring that all participants were able to engage.

The focus of the study was making services more equitable, and it therefore does not address other aspects of service design, such as the need for sustainable funding or the dilemmas around the content of prehabilitation programmes [6, 9]. With these limitations in mind, such considerations should not be interpreted as a rigid checklist but as guiding principles to inform service development and improvement.

This study provides consensus‐based considerations for equitable prehabilitation before cancer surgery which are grounded in the real‐world experience of service users and prehabilitation professionals. We encourage prehabilitation services to consider whether, and how, to implement these considerations to meet the needs of their populations.

## Supporting information


**Appendix S1.** Complete ACCORD Checklist.

**Appendix S2.** Summary list of considerations.

**Appendix S3.** Facilitation guide.

**Appendix S4.** Interim and final rankings of considerations.

**Appendix S5.** Plain English summary document.

## References

[1] Banugo P , Amoako D . Prehabilitation. BJA Educ 2017; 17: 401–405. 10.1093/bjaed/mkx032.

[2] Molenaar CJ , van Rooijen SJ , Fokkenrood HJ , Roumen RM , Janssen L , Slooter GD . Prehabilitation versus no prehabilitation to improve functional capacity, reduce postoperative complications and improve quality of life in colorectal cancer surgery. Cochrane Database Syst Rev 2022; 5: CD013259. 10.1002/14651858.cd013259.pub2. 35588252 PMC9118366

[3] Driessens H , Wijma AG , Buis CI , Nijkamp MW , Nieuwenhuijs‐Moeke GJ , Klaase JM . Prehabilitation: tertiary prevention matters. Br J Surg 2024; 111: znae028. 10.1093/bjs/znae028. 38436470 PMC10910596

[4] Stewart H , Zhang X , Hirst L , et al. Prehabilitation before cancer surgery in the UK National Health Service: what services exist, and how do they address health inequalities? PLoS One 2026; 21: e0336005. 10.1371/journal.pone.0336005. 42096417 PMC13152163

[5] van der Velde M , van der Leeden M , Geleijn E , Veenhof C , Valkenet K . What moves patients to participate in prehabilitation before major surgery? A mixed methods systematic review. Int J Behav Nutr Phys Act 2023; 20: 75. 10.1186/s12966-023-01474-6. 37344902 PMC10286498

[6] Stewart H , Stanley S , Zhang X , et al. The inequalities and challenges of prehabilitation before cancer surgery: a narrative review. Anaesthesia 2025; 80: 75–84. 10.1111/anae.16502. 39775660 PMC11744418

[7] Rampal T , Tribe S . Barriers to implementation of prehabilitation. Br J Hosp Med (Lond) 2025; 86: 1–24. 10.12968/hmed.2024.0817. 41284244

[8] Sontag AF , Kiselev J , Schaller SJ , Spies C , Rombey T . Facilitators and barriers to the implementation of prehabilitation for frail patients into routine health care: a realist review. BMC Health Serv Res 2024; 24: 192. 10.1186/s12913-024-10665-1. 38350947 PMC10863196

[9] Macmillan Cancer Support . Prehabilitation for people with cancer: clinical and implementation guidelines. London: Macmillan Cancer Support, 2025. https://www.macmillan.org.uk/healthcare‐professionals/cancer‐pathways/prehabilitation.

[10] Wareing L , Hirst Y , Shelton C , et al. Defining criteria for quality and equity in prehabilitation services before cancer surgery: a Delphi study informed by lived and professional experience. Eur J Cancer Care (Engl) 2025; 2025: 9308284. 10.1155/ecc/9308284.

[11] Hadley C , Zhang X , Stewart H , et al. Addressing inequities in the design and delivery of prehabilitation in the UK: case studies of challenges, complexities and good practice. Anaesthesia 2026. 10.1111/anae.70286. PMC13659788 42473333

[12] Gattrell WT , Logullo P , van Zuuren EJ , et al. ACCORD (ACcurate COnsensus reporting document): a reporting guideline for consensus methods in biomedicine developed via a modified Delphi. PLoS Med 2024; 21: e1004326. 10.1371/journal.pmed.1004326. 38261576 PMC10805282

[13] Harvey N , Holmes CA . Nominal group technique: an effective method for obtaining group consensus. Int J Nurs Pract 2012; 18: 188–194. 10.1111/j.1440-172x.2012.02017.x. 22435983

[14] McMillan SS , King M , Tully MP . How to use the nominal group and Delphi techniques. Int J Clin Pharmacol 2016; 38: 655–662. 10.1007/s11096-016-0257-x. PMC4909789 26846316

[15] McCormack LA , Treiman K , Rupert D , et al. Measuring patient‐centered communication in cancer care: a literature review and the development of a systematic approach. Soc Sci Med 2011; 72: 1085–1095. 10.1016/j.socscimed.2011.01.020. 21376443

[16] West MA , Carli F , Grocott MPW . Editorial: personalised multimodal prehabilitation in cancer. Front Oncol 2022; 12: 1086739. 10.3389/fonc.2022.1086739. 36505868 PMC9728579

[17] Paterson C , Roberts C , Toohey K , McKie A . Prostate cancer prehabilitation and the importance of multimodal interventions for person‐centred care and recovery. Semin Oncol Nurs 2020; 36: 151048. 10.1016/j.soncn.2020.151048. 32709485

[18] Fuchs TI , Pfab C , Kiselev J , Schaller SJ , Spies C , Rombey T . Barriers and facilitators to the implementation of prehabilitation for elderly frail patients prior to elective surgery: a qualitative study with healthcare professionals. BMC Health Serv Res 2024; 24: 536. 10.1186/s12913-024-10993-2. 38671446 PMC11046874

[19] Moore J , Merchant Z , Rowlinson K , et al. Implementing a system‐wide cancer prehabilitation programme: the journey of greater Manchester's 'Prehab4cancer. Eur J Surg Oncol 2021; 47: 524–532. 10.1016/j.ejso.2020.04.042. 32439265

[20] Bristow Z , Neck C , Cullum D , et al. Evaluating the Success of the Pioneering Prehab4Cancer (P4C) Programme Using innovative, Collaborative Methodology. https://gmcancer.org.uk/wp‐content/uploads/2022/10/71.‐Prehab4Cancer‐A1‐v3.pdf.

[21] Bonus CG , Hatcher D , Northall T , Montayre J . Enhancing culturally responsive care in perioperative settings for older adult patients: a qualitative interview study. Int J Nurs Stud 2025; 161: 104925. 10.1016/j.ijnurstu.2024.104925. 39566303

[22] Buckingham N . NHS Workforce in a Nutshell. London: The King's Fund, 2025. https://www.kingsfund.org.uk/insight‐and‐analysis/data‐and‐charts/nhs‐workforce‐nutshell.

