# Supplementary material for: Improving equity in prehabilitation before cancer surgery: consensus‐based considerations for leaders and practitioners: a nominal group technique study*
Source: Anaesth Rep. 2026 Jul 14;14(2):e70085. doi: 10.1002/anr3.70085 (PMC13369003; doi:10.1002/anr3.70085)
Supplement: Supplementary file 1 — Appendix S1. Complete ACCORD Checklist. [file ANR3-14-e70085-s003.pdf]

ACCORD
Accurate Consensus Reporting Document

Appendix S1: Complete ACCORD Checklist

Improving equity in prehabilitation before cancer surgery: consensus considerations for leaders and practitioners - a nominal group technique study

Zhang X, Ashmore L, Hadley C, et al. *Anaesthesia Reports* 2026.

Adapted from: Gattrell WT, Logullo P, van Zuuren EJ, et al. ACCORD (ACcurate COnsensus Reporting Document): A reporting guideline for consensus methods in biomedicine developed via a modified Delphi. *PLoS Med* 2024; 21: e1004326. doi: 10.1371/journal.pmed.1004326.

Item No.	Section	Checklist Item	Notes
T1	**Title**	Identify the article as reporting a consensus exercise and state the consensus methods used in the title.	Included in title
I1	**Introduction**	Explain why a consensus exercise was chosen over other approaches.	Included in Manuscript (Introduction) - Role of consensus where evidence is uncertain and multiple perspectives required.
I2		State the aim of the consensus exercise, including its intended audience and geographical scope (national, regional, global).	Included in Manuscript (Introduction) - Intended audience - strategic leads, service leads, practitioners. Geographical scope - UK NHS
I3		If the consensus exercise is an update of an existing document, state why an update is needed, and provide the citation for the original document.	N/A
M1	**Methods** Registration	If the study or study protocol was prospectively registered, state the registration platform and provide a link. If the exercise was not registered, this should be stated.	Included in Manuscript (Methods) - Institutional registration via ethical approval process (June 2025).<br>Not publicly registered.
M2	Selection of SC and/or panellists	Describe the role(s) and areas of expertise or experience of those directing the consensus exercise.	Included in Manuscript (Methods) - Selection by the PARITY team, had been running a national mixed methods study of prehabilitation since June 2022
M3		Explain the criteria for panellist inclusion and the rationale for panellist numbers. State who was responsible for panellist selection.	Included in Manuscript (Methods) - Involvement was based on role in prehabilitation (i.e. professional or lived-experience expertise)
M4		Describe the recruitment process (how panellists were invited to participate).	Included in Manuscript (Methods) - Invitation was via relevant professional groups and

			involvement in earlier stages of the study. Invitations were sent via email.
M5		Describe the role of any members of the public, patients or carers in the different steps of the study.	Included in Manuscript (Methods) - Involved as group members, with a structured process to support meaningful participation.
M6	Preparatory research	Describe how information was obtained prior to generating items or other materials used during the consensus exercise.	Included in Manuscript (Methods) - Based on identification in prior phases of the project.
M7		Describe any systematic literature search in detail, including the search strategy and dates of search or the citation if published already.	N/A
M8		Describe how any existing scientific evidence was summarised and if this evidence was provided to the panellists.	Included in Manuscript (Methods) - Considerations (Appendix S2) sent to participants, two weeks in advance of the event.
M9	Assessing consensus	Describe the methods used and steps taken to gather panellist input and reach consensus (for example, Delphi, RAND-UCLA, nominal group technique).	Included in Manuscript (Methods) - Nominal group technique, with idea generation based on prior phases of the project.
M10		Describe how each question or statement was presented and the response options. State whether panellists were able to or required to explain their responses, and whether they could propose new items.	Included in Manuscript (Methods) - Considerations (Appendix S2) were sent to participants, who were asked to choose their 'top three' and 'bottom three' options, and explain these during the workshop.
M11		State the objective of each consensus step.	Included in Manuscript (Methods) - Experience to prioritise considerations based on their perceived impact and feasibility, with the aim of informing a best practice guide for equitable prehabilitation services
M12		State the definition of consensus (for example, number, percentage, or categorical rating, such as 'agree' or 'strongly agree') and explain the rationale for that definition.	Included in Manuscript (Methods) - Ranking process, with results agreed in the final plenary session<br>Majority vote would have been used if agreement had not been unanimous.
M13		State whether items that met the prespecified definition of consensus were included in any subsequent voting rounds.	N/A
M14		For each step, describe how responses were collected, and whether responses were collected in a group setting or individually.	Included in Manuscript (Methods) - Group setting, based on rank-order, via an online facilitation platform.
M15		Describe how responses were processed and/or synthesised.	Included in Manuscript (Methods) - Based on average rankings between groups.

M16		Describe any piloting of the study materials and/or survey instruments.	The wording of the considerations was agreed Included in Manuscript (Methods) - by the PARITY Study team following review of findings of the other phases of the project
M17		If applicable, describe how feedback was provided to panellists at the end of each consensus step or meeting.	Included in Manuscript (Methods) - A preliminary ranked list derived from the average rankings of the prior step.
M18		State whether anonymity was planned in the study design. Explain where and to whom it was applied and what methods were used to guarantee anonymity.	Included in Manuscript (Methods) - The ranking was based on group work, which was not anonymous.
M19		State if the steering committee was involved in the decisions made by the consensus panel.	Included in Manuscript (Methods) - The PARITY study team facilitated the workshop; they did not have voting rights.
M20	Participation	Describe any incentives used to encourage responses or participation in the consensus process.	Public voice participants were reimbursed for their time according to NIHR-suggested rates.
M21		Describe any adaptations to make the surveys/meetings more accessible.<br>.	All participants received reimbursement of expenses; both online and in-person attendance was available.
R1	Results	State when the consensus exercise was conducted. List the date of initiation and the time taken to complete each consensus step, analysis, and any extensions or delays in the analysis.	A full day workshop in July 2025, with two weeks for preparatory work.
R2		Explain any deviations from the study protocol, and why these were necessary.	N/A
R3		For each step, report quantitative (number of panellists, response rate) and qualitative (relevant socio-demographics) data to describe the participating panellists.	Included in the manuscript (results section)
R4		Report the final outcome of the consensus process as qualitative (for example, aggregated themes from comments) and/or quantitative (for example, summary statistics, score means, medians and/or ranges) data.	Included in the manuscript (Box 1 and Table 2)
R5		List any items or topics that were modified or removed during the consensus process. Include why and when in the process they were modified or removed.	N/A
D1	Discussion	Discuss the methodological strengths and limitations of the consensus exercise.	Included in the manuscript (discussion section)
D2		Discuss whether the recommendations are consistent with any pre-existing literature and, if not, propose reasons why this process may have arrived at alternative conclusions.	Included in the manuscript (discussion section)
O1	Other information	List any endorsing organisations involved and their role.	N/A
O2		State any potential conflicts of interests, including among those directing the consensus study and panellists. Describe how conflicts of interest were managed.	Included in the manuscript (acknowledgements section)

O3		State any funding received and the role of the funder.	Included in the manuscript (acknowledgements section)

For more information see: https://www.ismpp.org/accord
