# Supplementary material for: Improving equity in prehabilitation before cancer surgery: consensus‐based considerations for leaders and practitioners: a nominal group technique study*
Source: Anaesth Rep. 2026 Jul 14;14(2):e70085. doi: 10.1002/anr3.70085 (PMC13369003; doi:10.1002/anr3.70085)
Supplement: Supplementary file 2 — Appendix S2. Summary list of considerations. [file ANR3-14-e70085-s004.pdf]

Appendix S2: Summary list of considerations

Improving equity in prehabilitation before cancer surgery: consensus considerations for leaders and practitioners - a nominal group technique study

Zhang X, Ashmore L, Hadley C, et al. *Anaesthesia Reports* 2026.

The wording in the ‘consideration text’ column is the original wording used for the workshop. The final version (in the main manuscript) includes some minor changes for consistency of style, which were agreed with the group following the workshop.

<table>
<tr><td colspan="5">In advance of the workshop on 30th July, please read the below recommendations for equitable delivery of prehabilitation before cancer surgery, and decide which you think are the three most important ('top 3'), and the three least important ('bottom 3') - you can mark these on this sheet with a tick in the appropriate column as well as making any notes. You may wish to consider both their impact (how much good you think they would do), and their feasibility (whether you think services would be able to make them happen). The code number is just an identifier which you can use as a shorthand on the day. If you have any questions about this activity, feel free to email [the PARITY Study email]</td></tr>
<tr><th>Code</th><th>Consideration Text</th><th>Top 3?</th><th>Bottom 3?</th><th>Notes</th></tr>
<tr><td>1</td><td>Regardless of where they live, there is a conversation to understand how patient’s prehabilitation care plan and subsequently cancer surgery will affect their life.</td><td></td><td></td><td></td></tr>
<tr><td>2</td><td>Interpreters are always provided for those who need them (including for sensory impairment) at each appointment.</td><td></td><td></td><td></td></tr>
<tr><td>3</td><td>The care team is trained to improve their understanding of equality and diversity issues.</td><td></td><td></td><td></td></tr>
<tr><td>4</td><td>The service has a group of ‘key contacts’ to provide advice for caring for patients with protected characteristics and vulnerable people when they are referred.</td><td></td><td></td><td></td></tr>
<tr><td>5</td><td>Prehabilitation teams should accommodate patients who wish to be cared for by people of their own gender</td><td></td><td></td><td></td></tr>
</table>

6	*Prehabilitation teams should signpost community resources (e.g., leisure centres) which have gender-specific facilities (e.g., women-only gyms)*			
7	*Prehabilitation literature (e.g., information leaflets) should be available in languages which are commonly-used by the local population*			
8	*Prehabilitation staff who provide dietary advice should be trained on cultural and religious dietary conventions and requirements.*			
9	*Prehabilitation teams should signpost retailers which supply exercise clothing appropriate for patients' religious / cultural preferences*			
10	*Patients' relatives and carers should be included in prehabilitation appointments, if the patient expresses a preference for this to take place*			
11	*Patients with learning disabilities should be enabled to participate in prehabilitation, in collaboration with learning disability nurses / teams*			
12	*Prehabilitation services should provide travel / transport services, to enable attendance by patients who are less able to travel independently*			
13	*Prehabilitation services should provide a wide range of appointment times, to enable participation by patients who have time-bound commitments (e.g., work, caring responsibilities)*			
14	*Prehabilitation should take place in facilities which are fully accessible to patients with restricted mobility*			
15	*Prehabilitation activities should be adapted to facilitate the participation of people living with disabilities*			

16	*Where digital data entry is needed (e.g., during screening), patients should be offered support to complete this*			
17	*Where patients are unable or less able to travel to attend prehabilitation services, home visits should be offered.*			
18	*Prehabilitation teams should signpost patients to financial support services (e.g., charities, credit unions)*			
19	*Prehabilitation services should reimburse / provide travel expenses to facilitate the attendance of patients who are less able to afford travel*			
20	*Prehabilitation services should provide food vouchers to support patients who are less able to afford food to engage with dietary recommendations*			
21	*Prehabilitation services should negotiate discounted access to leisure facilities, to facilitate patients' participation in exercise*			
22	*Prehabilitation services should loan equipment (e.g., exercise equipment, digital equipment) to patients to facilitate their engagement in prehabilitation*			
23	*Prehabilitation activities should be made available using digital technologies (e.g., videoconferencing), to facilitate participation without the need for in-person attendance*			
24	*Prehabilitation activities should be made available using telephone, to facilitate participation without the need for in-person attendance*			
25	*Where a prehabilitation service covers a large geographical area, activities should be spread across that area (e.g., using local healthcare facilities / community leisure centres) to minimise patient travel distances.*			

26	*Prehabilitation teams should make referrals to support services (e.g., charities, social services, social prescribers) where a need is identified*			
27	*Prehabilitation activities should be made available on a face-to-face basis, to facilitate participation without access to digital / telephone facilities*			
28	*Prehabilitation literature (e.g., patient information leaflets) should be made available in paper form, to facilitate engagement by patients without access to digital facilities*			
29	*Prehabilitation services should arrange local accommodation to facilitate the participation of patients from out of area.*			
30	*Prehabilitation services should refer patients who are from out of area to local prehabilitation programmes to facilitate their participation.*			
31	*Prehabilitation services should facilitate peer support between patents*			
32	*Appointments to attend prehabilitation activities should be available to suit diverse religious / cultural calendars*			
33	*Digital resources (e.g., videos, images) should include subtitles / metadata*			
34	*Professional communication related to prehabilitation should be shared/integrated with other professionals involved in the patient's care (e.g., primary care, surgical team)*			
35	*Prehabilitation services should have a clear and dedicated means of contact for patients (e.g., telephone hotline)*			

36	*Prehabilitation services should be made available on a 'systemwide' level (i.e., across the geographical footprint of a healthcare organisation).*			
37	*Prehabilitation services should include outreach to contact patients who do not appear to be engaging, and identify barriers to participation*			
38	*Prehabilitation services should be prepared to adapt the way they are delivered to suit the needs of individual patients, and facilitate their participation*			
39	*Prehabilitation staff who provide dietary advice should accommodate patients' preferences when developing nutrition plans*			
40	*Where patients are expected to undertake administration to participate in prehabilitation (e.g., booking appointments, arranging transport), they should be offered support to complete this*			
41	*Prehabilitation teams should acknowledge and normalise that patients may find the activities involved in prehabilitation challenging (e.g., due to physical fitness and / or symptoms)*			
42	*Prehabilitation teams should encourage patients to engage in physical activity based on their usual everyday activities (e.g., walking the dog)*			
