# Supplementary material for: Improving equity in prehabilitation before cancer surgery: consensus‐based considerations for leaders and practitioners: a nominal group technique study*
Source: Anaesth Rep. 2026 Jul 14;14(2):e70085. doi: 10.1002/anr3.70085 (PMC13369003; doi:10.1002/anr3.70085)
Supplement: Supplementary file 3 — Appendix S3. Facilitation guide. [file ANR3-14-e70085-s001.pdf]

Appendix S3: Facilitation Guide

Improving equity in prehabilitation before cancer surgery: consensus considerations for leaders and practitioners - a nominal group technique study

Zhang X, Ashmore L, Hadley C, et al. *Anaesthesia Reports* 2026.

Please note that we use the terms 'recommendations' and 'statements' to describe the 42 considerations for more equitable prehabilitation before cancer surgery in the below document. These terms were changed to 'considerations' following a discussion with the group, and we use that term in the main manuscript.

Facilitation Guide for PARITY consensus event

The purpose of this event is to put the 42 PARITY recommendations into order. This is to help prioritise their implementation in healthcare practice.

Attendees have already been sent a list of the 42 recommendations, and invited to read through them and pick the three that they think are most important, and the three that they think are least important. They have been asked to consider *impact* and *feasibility of implementation* when making these choices.

There will be two rounds of discussion at the event, in two small groups of 5-8 participants. Some participants will be online.

The 'end product' of each round should be an ordered list of recommendations, with the highest priority at the top, and the lowest priority at the bottom.

The first round of discussions will commence without any pre-ordering.

The second round of discussions will commence with the recommendations ordered according to an average of the first round of discussions.

The average ordering from the second round of discussions will be presented at the final plenary, where further changes can be made by consensus of the group.

A flow chart of the day is in Fig. 1:

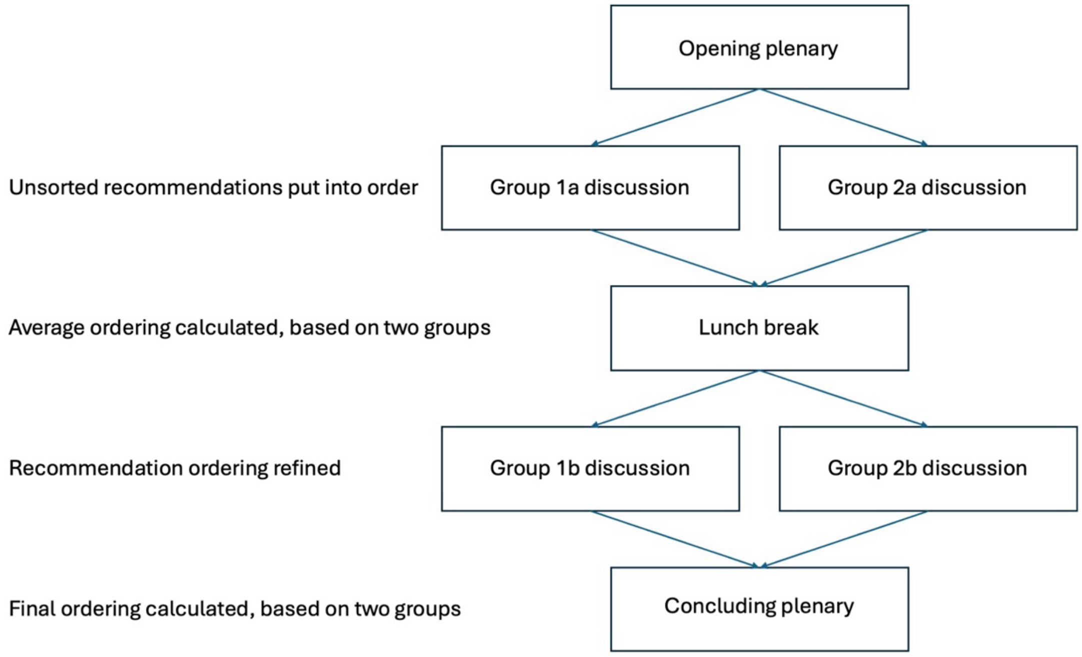

Figure 1: event flow chart

Guidelines for facilitating small group discussions

There will be a mix of participants, including patient / public representatives and professionals. It is possible that some of these people will feel uncomfortable speaking frankly in front of others, so the key role of the facilitators is to ensure that everyone is heard. The morning group lasts for 1hr 40min (plus 5 min to move to the room). The afternoon group lasts 1hr (plus 5 min to move to the room).

Process for the morning discussion (feel free to amend to meet participants' needs):

- Start with brief intros – just a name (+/- job title or role) and "one reason why they are interested in this topic" (stick to 'one reason' to avoid long intros and keep things balanced).
- Invite participants to share their top & bottom three recommendations.
    - As 'top threes' are suggested, move them towards the green side of the board.
    - As 'bottom threes' are suggested, move them towards the orange side of the board.
    - Encourage some explanation / discussion as to why they have chosen these recommendations
- Once everyone has had some 'airtime', work with the group to collaboratively put the recommendations into order.
    - Remind them to consider both consider *impact* and *feasibility of implementation* when making these choices.
- All recommendations need to be ordered by the end of the discussion. One way to encourage a choice is to ask 'is this recommendation more or less important than [pick another recommendation] – this will allow you to place it above or below that other recommendation.
- Top Tips:
    - Keep an eye on the time – the 'airtime' should last around 40 mins, leaving an hour for discussion.
    - Remember to include online participants – they are easy to miss in a mixed group.
    - You may need to use some facilitation skills to encourage quieter group members to participate (and ask dominant members to give them some space).

Process for the afternoon discussion (feel free to amend to meet participants' needs):

- The group will have been mixed up - start with brief intros as per the morning.
- Invite participants to look at the ordered list, and consider if any of the recommendations need to be moved.
- Go round each member of the group, and ask if they would like to suggest any changes. If any recommendations are mentioned, you can move them to the side of the board, as a prompt for discussion.
- Once everyone has had some 'airtime', work with the group to collaboratively refine the order of recommendations (if needed)
    - Remind them to consider both consider *impact* and *feasibility of implementation* when making these choices.
- Top Tips:
    - Keep an eye on the time – the 'airtime' should last around 30 mins, leaving 30 mins for ordering.
    - Remember to include online participants – they are easy to miss in a mixed group.
    - You may need to use some facilitation skills to encourage quieter group members to participate (and ask dominant members to give them some space).

Event timings / programme

09:30 - 10:30	Coffee and registration

10:30-10:50	Intro
10:55 - 12:20	Discussion Group 1
12:20 - 13:05	Lunch
13:05 - 13:15	Recap
13:20 - 14:45	Discussion Group 2
14:50 - 15:20	Final Plenary
15:20 - 15:30	Summing up

Using Miro

To make the process equally visible to online and in-person attendees, we will use Miro, an online whiteboard, to display and move the recommendations.

Before the first session, the Miro whiteboard looks like the image in Fig 2 (zoomed all the way out).

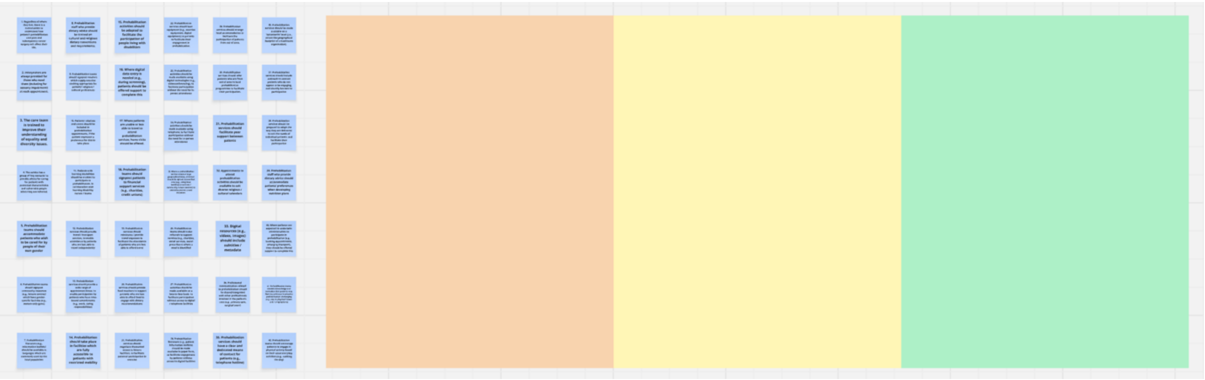

Figure 2: The PARITY consensus Miro Whiteboard

The blue squares on the left are the PARITY recommendations on ‘post-it notes’ – zoomed in examples are shown in Figure 3. You will need to zoom in during discussions so that participants can see what is being discussed.

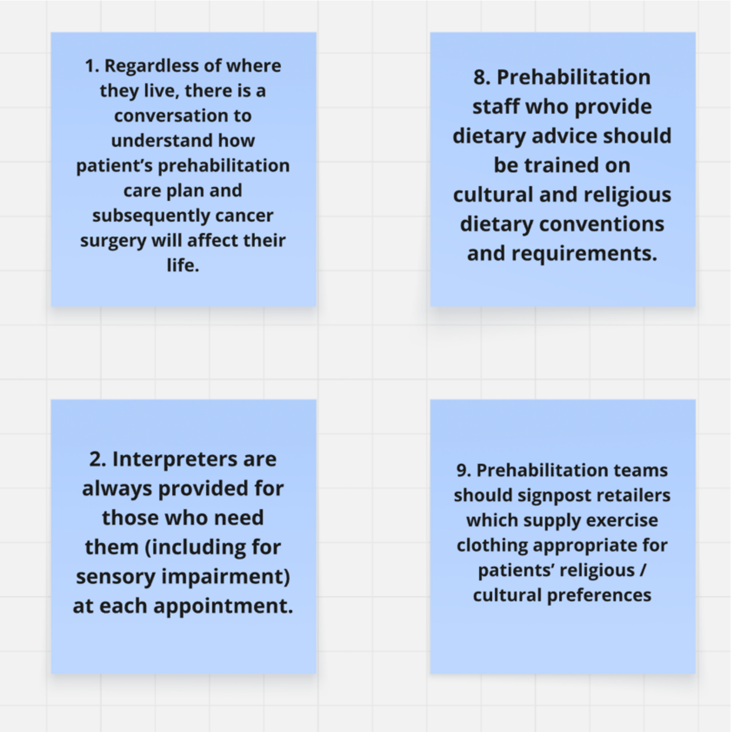

Figure 3: example recommendation statements.

As the recommendations are discussed, you can move them to a coloured area of the whiteboard (to the right of the zoomed-out screen). If someone nominates a 'top three', move it to the green area. If someone nominates a 'bottom three' move it to the orange area, and if something isn't mentioned, you can move it to the yellow area.

To move something, simply click and drag with the cursor (it will be highlighted with a blue box as shown in Fig 4.

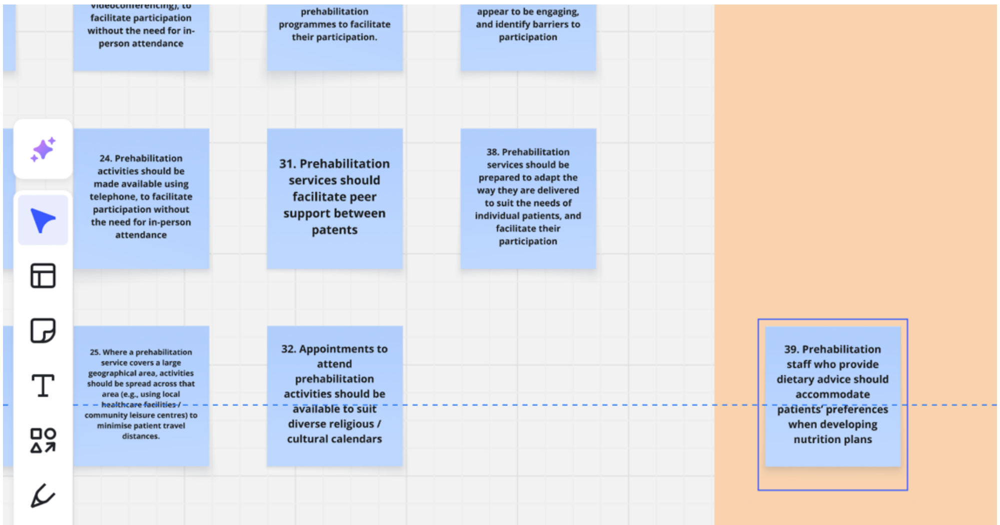

Figure 4: dragging a recommendation to a new location

By the end of each discussion, you must have a clear order visible on the Miro whiteboard, with the most important on the right, and the least important on the left. An example is shown in Fig. 5.

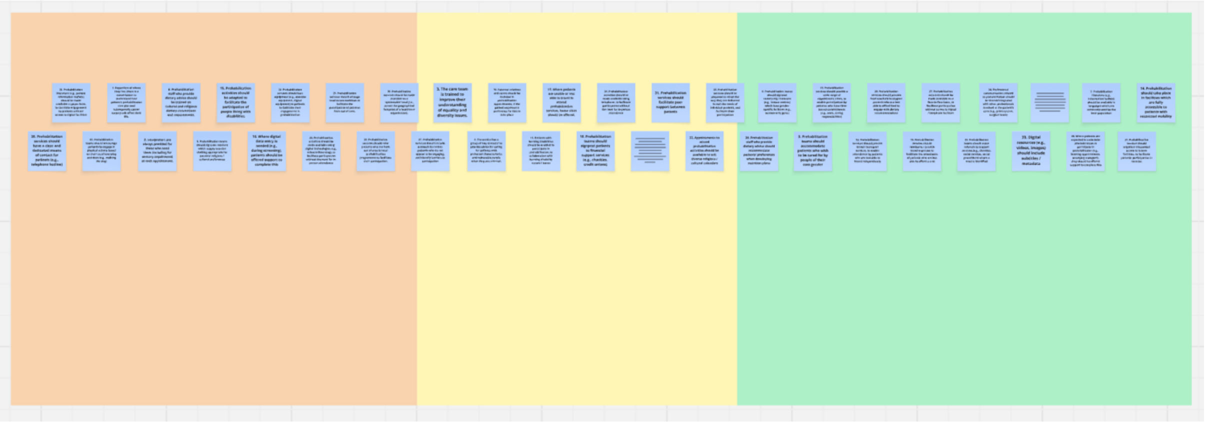

Figure 5: example of ordered statements
