# Supplementary material for: Improving equity in prehabilitation before cancer surgery: consensus‐based considerations for leaders and practitioners: a nominal group technique study*
Source: Anaesth Rep. 2026 Jul 14;14(2):e70085. doi: 10.1002/anr3.70085 (PMC13369003; doi:10.1002/anr3.70085)
Supplement: Supplementary file 4 — Appendix S4. Interim and final rankings of considerations. [file ANR3-14-e70085-s005.pdf]

Appendix S4: Interim and final rankings of considerations

Improving equity in prehabilitation before cancer surgery: consensus considerations for leaders and practitioners - a nominal group technique study

Zhang X, Ashmore L, Hadley C, et al. Anaesthesia Reports. 2026.

The wording in the 'consideration text' column is the original wording used for the workshop. The final version (in the main manuscript) includes some minor changes for consistency of style, which were agreed with the group following the workshop.

The 'average rank' columns are calculated based on dividing the sum of the group ranks by the number of small groups (2).

The 'consensus rank' column was agreed by the whole group in the final plenary session.

		Morning			Afternoon			Final Plenary
**Consideration text**	**Level of Implementation**	**Group 1A Rank**	**Group 1B Rank**	**Average Rank**	**Group 1B Rank**	**Group 2B Rank**	**Average Rank**	**Consensus Rank**
Regardless of where they live, there is a conversation to understand how patient's prehabilitation care plan and subsequently cancer surgery will affect their life.	Operational leaders	1	1	1	1	1	1	1
Prehabilitation services should be prepared to adapt the way they are delivered to suit the needs of individual patients, and facilitate their participation	Operational leaders	6	6	6	2	2	2	2
Prehabilitation services should be made available on a 'systemwide' level (i.e., across the geographical footprint of a healthcare organisation).	Strategic Leaders	10	3	6.5	3	3	3	3
Prehabilitation services should include outreach to contact patients who do not appear to be engaging, and identify barriers to participation	Operational leaders	12	2	7	4	6	5	4
Interpreters are always provided for those who need them (including for sensory impairment) at each appointment.	Operational leaders	6	10	8	5	7	6	5
The care team is trained to improve their understanding of equality and diversity issues.	Operational leaders	3	9	6	8	5	7	6

Where a prehabilitation service covers a large geographical area, activities should be spread across that area (e.g., using local healthcare facilities / community leisure centres) to minimise patient travel distances.	Strategic Leaders	9	7	8	6	8	7	7
Professional communication related to prehabilitation should be shared/integrated with other professionals involved in the patient's care (e.g., primary care, surgical team)	Strategic Leaders	14.5	5	9.75	10	4	7	8
Prehabilitation services should provide a wide range of appointment times, to enable participation by patients who have time-bound commitments (e.g., work, caring responsibilities)	Operational leaders	13	4	8.5	7	9	8	9
Prehabilitation staff who provide dietary advice should be trained on cultural and religious dietary conventions and requirements.	Operational leaders	6	13	9.5	9	10	10	10
Prehabilitation teams should acknowledge and normalise that patients may find the activities involved in prehabilitation challenging (e.g., due to physical fitness and / or symptoms)	Practitioners	14.5	8	11.25	11	11	11	11
Patients' relatives and carers should be included in prehabilitation appointments, if the patient expresses a preference for this to take place	Practitioners	11	19	15	12	12	12	12
Prehabilitation teams should encourage patients to engage in physical activity based on their usual everyday activities (e.g., walking the dog)	Practitioners	14.5	16	15.25	13	13	13	13
Prehabilitation activities should be adapted to facilitate the participation of people living with disabilities	Practitioners	6	28	17	14	14	14	14
Where patients are expected to undertake administration to participate in prehabilitation (e.g., booking appointments, arranging transport), they should be offered support to complete this	Operational leaders	19.5	15	17.25	15	15	15	15
Prehabilitation should take place in facilities which are fully accessible to patients with restricted mobility	Operational leaders	26	11	18.5	16	16	16	16

Where digital data entry is needed (e.g., during screening), patients should be offered support to complete this	Operational leaders	14.5	24	19.25	17	17	17	17
Prehabilitation services should negotiate discounted access to leisure facilities, to facilitate patients' participation in exercise	Strategic Leaders	17	23	20	18	18	18	18
Digital resources (e.g., videos, images) should include subtitles / metadata	Operational leaders	6	34	20	19	19	19	19
Prehabilitation services should loan equipment (e.g., exercise equipment, digital equipment) to patients to facilitate their engagement in prehabilitation	Strategic Leaders	34	36	35	20	38	29	20
Prehabilitation activities should be made available using digital technologies (e.g., videoconferencing), to facilitate participation without the need for in-person attendance	Strategic Leaders	23	20	21.5	21	20	21	21
Prehabilitation teams should make referrals to support services (e.g., charities, social services, social prescribers) where a need is identified	Operational leaders	29	17	23	22	21	22	22
Prehabilitation teams should signpost community resources (e.g., leisure centres) which have gender-specific facilities (e.g., women-only gyms)	Operational leaders	11	37	24	23	22	23	23
Prehabilitation literature (e.g., patient information leaflets) should be made available in paper form, to facilitate engagement by patients without access to digital facilities	Operational leaders	22	26	24	24	23	24	24
Prehabilitation teams should signpost patients to financial support services (e.g., charities, credit unions)	Operational leaders	31	18	24.5	25	24	25	25
Prehabilitation activities should be made available on a face-to-face basis, to facilitate participation without access to digital / telephone facilities	Strategic Leaders	24	25	24.5	26	25	26	26
Prehabilitation services should refer patients who are from out of area to local prehabilitation programmes to facilitate their participation.	Operational leaders	18	31	24.5	27	26	27	27
Prehabilitation services should facilitate peer support between patents	Operational leaders	35	14	24.5	28	27	28	28

Prehabilitation services should reimburse / provide travel expenses to facilitate the attendance of patients who are less able to afford travel	Strategic Leaders	38	12	25	29	28	29	29
Prehabilitation staff who provide dietary advice should accommodate patients' preferences when developing nutrition plans	Practitioners	19.5	32	25.75	30	29	30	30
Prehabilitation literature (e.g., information leaflets) should be available in languages which are commonly-used by the local population	Operational leaders	27	27	27	31	30	31	31
Patients with learning disabilities should be enabled to participate in prehabilitation, in collaboration with learning disability nurses / teams	Strategic Leaders	25	29	27	32	31	32	32
Prehabilitation activities should be made available using telephone, to facilitate participation without the need for in-person attendance	Strategic Leaders	33	21	27	33	32	33	33
Prehabilitation services should provide food vouchers to support patients who are less able to afford food to engage with dietary recommendations	Strategic Leaders	36	22	29	34	33	34	34
Appointments to attend prehabilitation activities should be available to suit diverse religious / cultural calendars	Operational leaders	21	41	31	35	34	35	35
Prehabilitation services should have a clear and dedicated means of contact for patients (e.g., telephone hotline)	Operational leaders	32	30	31	36	35	36	36
The service has a group of 'key contacts' to provide advice for caring for patients with protected characteristics and vulnerable people when they are referred.	Operational leaders	28	38	33	37	36	37	37
Prehabilitation teams should accommodate patients who wish to be cared for by people of their own gender	Operational leaders	30	39	34.5	38	37	38	38
Prehabilitation services should provide travel / transport services, to enable attendance by patients who are less able to travel independently	Strategic Leaders	38	33	35.5	39	39	39	39
Where patients are unable or less able to travel to attend prehabilitation services, home visits should be offered.	Strategic Leaders	38	35	36.5	40	40	40	40

Prehabilitation teams should signpost retailers which supply exercise clothing appropriate for patients' religious / cultural preferences	Operational leaders	40	40	40	41	41	41	41
Prehabilitation services should arrange local accommodation to facilitate the participation of patients from out of area.	Strategic Leaders	41	42	41.5	42	42	42	42
