# Supplementary material for: Improving equity in prehabilitation before cancer surgery: consensus‐based considerations for leaders and practitioners: a nominal group technique study*
Source: Anaesth Rep. 2026 Jul 14;14(2):e70085. doi: 10.1002/anr3.70085 (PMC13369003; doi:10.1002/anr3.70085)
Supplement: Supplementary file 5 — Appendix S5. Plain English summary document. [file ANR3-14-e70085-s002.pdf]

Health & Medicine | Lancaster University

PARITY

Prehabiliation for Cancer Surgery: Quality and Inequality

Improving equitable access to prehabilitation before cancer surgery: Good practice considerations for leaders and practitioners.

What is the PARITY study?

The PARITY study aims to reduce unwarranted variation and inequity in prehabilitation before cancer surgery by combining consensus-building, national mapping, and in-depth case studies. In the first part of the study, we sought to build agreement among patients, carers, and professionals on the aims, objectives, and values of prehabilitation, clarifying what it is, what it should achieve, and how it should be delivered.

The second part of the study involved mapping prehabilitation services available to patients preparing for cancer surgery across the UK to understand where and how they operate, who provides them, and how inequalities are addressed. The third part of the study involved detailed case studies of six diverse services to understand patient experience and real-world practice, and identify strategies for tackling inequalities.

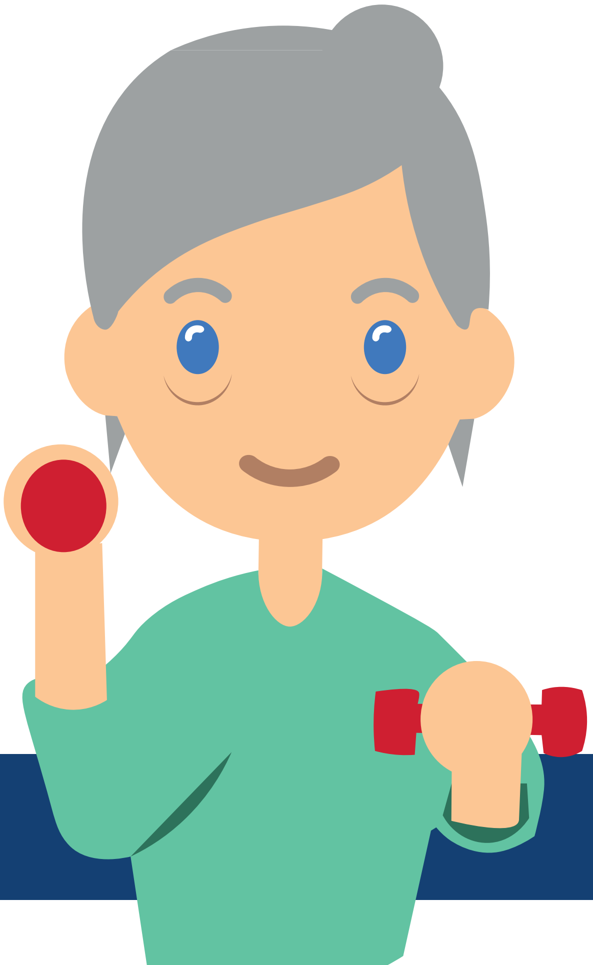

This work was funded by the National Institute for Health and Care Research (NIHR) Health Services and Delivery Research programme as part of the PARITY study (NIHR134282). The views expressed are those of the authors and not necessarily those of the NIHR or the Department of Health and Social Care.

v9, 07/26

What is prehabilitation?

Prehabilitation seeks to enhance a patient's fitness and preparedness before major interventions, such as surgery, by turning 'waiting time' into 'preparation time'. Typically it includes exercise, dietary, and psychological interventions (alone or in combination) within a behaviour change framework. It aims to speed up recovery, reduce healthcare resource use, and foster an enhanced sense of control for patients.

Why good practice considerations are needed

Prehabilitation before cancer surgery is a complex intervention that requires patient engagement, time, and resources. Social determinants, such as socioeconomic status, ethnicity, and geographic location, are associated with different levels of participation in prehabilitation, resulting in 'intervention-generated inequalities'. Patients may also experience physical symptoms, competing treatment priorities, and logistical barriers to participation, while prehabilitation providers must navigate varied evidence, conflicting clinical guidance, and inconsistent funding. This makes delivering equitable prehabilitation challenging.

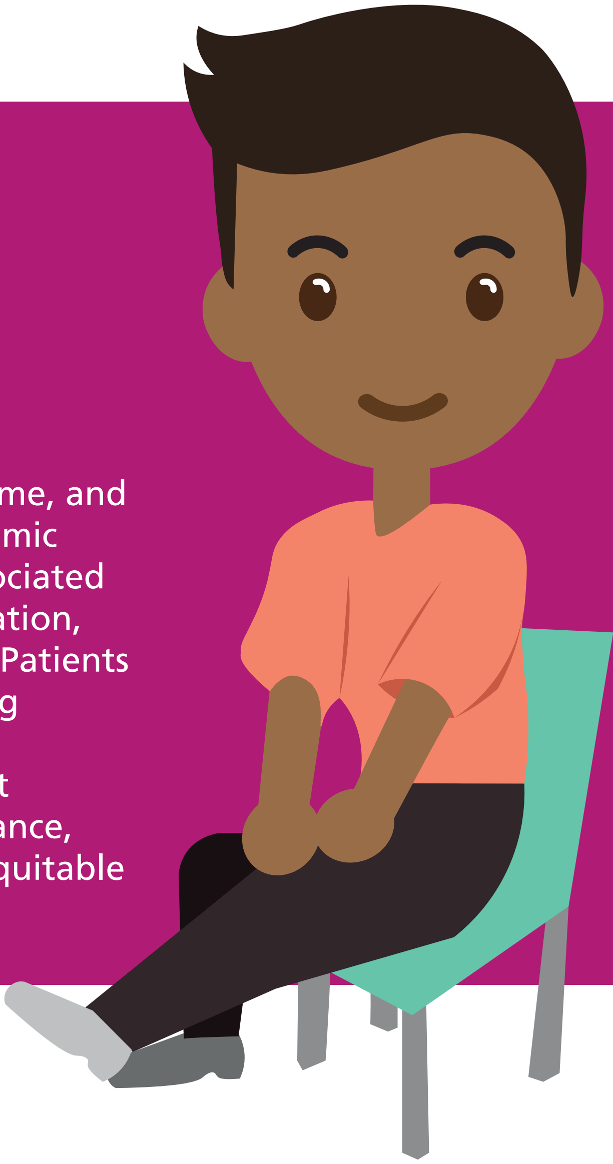

The Macmillan **Prehabilitation for Cancer: Clinical and Implementation Guidelines** (September 2025) acknowledge the importance of addressing inequalities, and state:

> **"We recommend that health inequalities should be considered in the design, delivery and implementation of prehabilitation services and the associated interventions to avoid exclusion."**
>
> **Macmillan**

In the PARITY study, we found that prehabilitation providers and healthcare leaders recognise many of the challenges of intervention generated inequalities and are already implementing measures to make participation in prehabilitation more equitable. However, it was not clear which of these were most effective, and whether strategies implemented in one service could be used in other services.

Consequently, in the final stages of our study, we held a consensus workshop with professional and patient representatives to examine ways for improving access to prehabilitation identified throughout the PARITY study.

We asked them to draw on their knowledge and experience and identify which ones were the most important, to inform a 'best practice guide' for prehabilitation services.

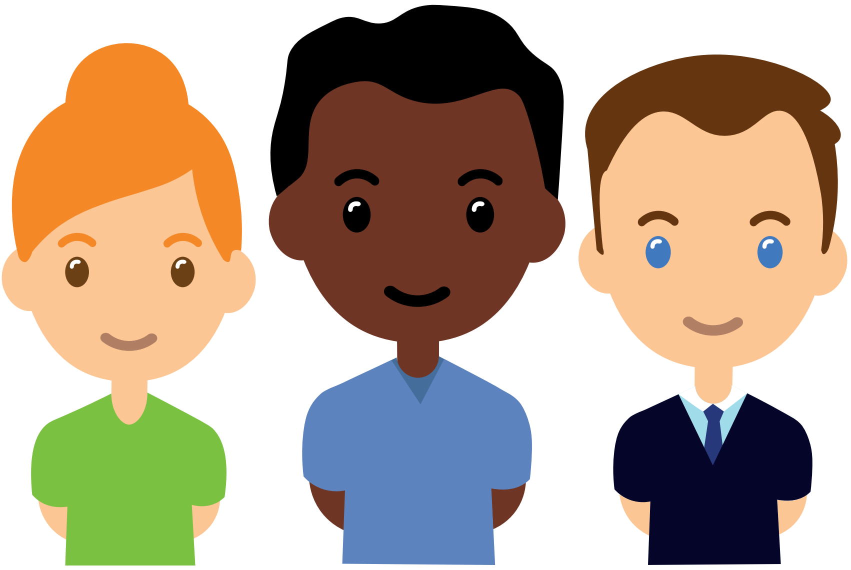

How was consensus workshop done?

Across its three sub-studies, the PARITY study identified 42 ways to promote equitable access to prehabilitation, with a focus on inclusivity, accessibility, and patient-centred care. We presented these to a panel of 15 people with expertise in prehabilitation, including patient and public representatives, prehabilitation practitioners, and strategic leaders, and invited them to identify which were the most important recommendations on the basis of impact and feasibility of implementation. We used a method called the nominal group technique, which involves rounds of small group work and full panel discussion, to ensure that everyone had the opportunity to speak.

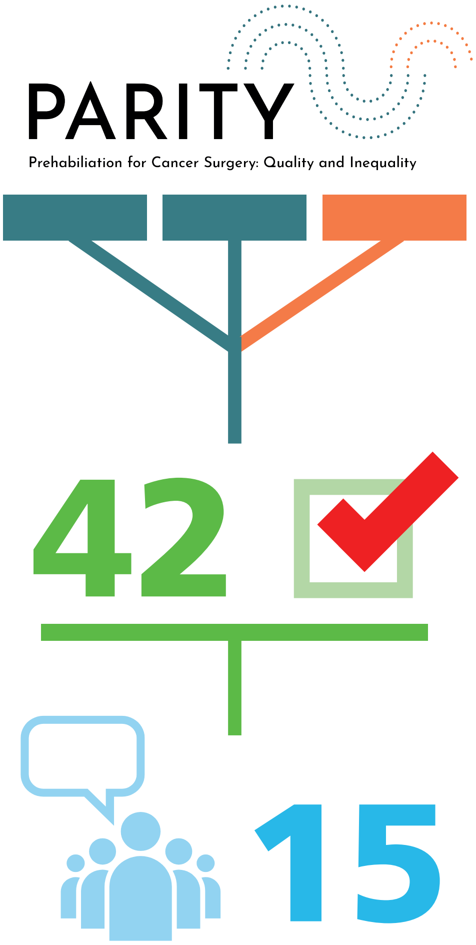

How should the good practice considerations be used?

We believe that these considerations will be useful to:

Strategic leaders (e.g. commissioners and directors - who can make decisions on the scope, reach and funding of a service, and make arrangements with external organisations)

Operational leaders (e.g., service leads - who can make decisions about how their prehabilitation service is delivered, and the resources / activities that are involved)

Practitioners (e.g. exercise specialists, health professionals - who can make decisions about how they work within their role in a prehabilitation service).

We recognise that some people may hold multiple roles simultaneously (e.g. a service lead may also deliver prehabilitation as a practitioner), and we also acknowledge that this list is not exhaustive – it is drawn from existing service designs, practices, guidance and opinions – so it should be viewed as a starting point for more equitable prehabilitation, not a definitive resource.

We would also caution against using it as a ‘tick box exercise’, and encourage prehabilitation services to consider whether, and how, to implement these considerations to meet the needs of their populations. Please note that these considerations only focus on making prehabilitation services more equitable, they do not address other aspects of service design such as funding, or what prehabilitation actually involves. In common with all health services, prehabilitation should fulfil some basic requirements, including:

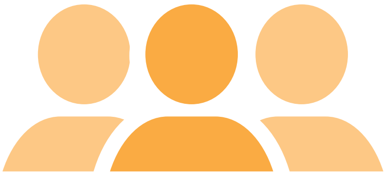

Have access to adequate staffing, funding and facilities

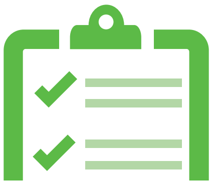

Participate in audit, evaluation and quality improvement

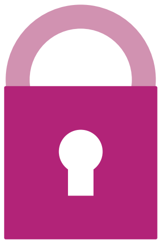

Offer privacy / confidentiality when required

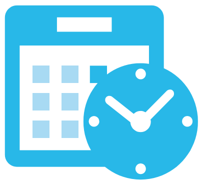

Offer appointments of an appropriate duration for patient needs

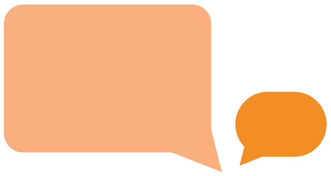

Communicate with service users in plain language

Which were the highest-ranked practice considerations?

The six considerations to be most important overall were:

There should be a conversation to understand how a patient's prehabilitation care plan and subsequently cancer surgery will affect their life

Prehabilitation services should be prepared to adapt the way they are delivered to suit the needs of individual patients, and facilitate their participation

Prehabilitation services should be made available on a 'systemwide' level (i.e., across the geographical footprint of a healthcare organisation)

Prehabilitation services should include outreach to contact patients who do not appear to be engaging, and identify barriers to participation

Interpreters should be provided for those who need them (including for sensory impairment) at each appointment

The care team should be trained to improve their understanding of equality and diversity issues

These good practice considerations relate to the following themes:

Communication & Personalisation:

Ensure patients understand how prehabilitation and surgery will affect their lives, adapt delivery to individual needs, and engage those not participating.

Accessibility & Equity:

Provide interpreters, accessible facilities, outreach, and support for administrative or digital tasks; offer varied appointment times and cultural/religious sensitivity.

Systemwide & Strategic Support:

Deliver prehabilitation across whole health systems, integrate communication with other healthcare providers, and offer face-to-face, digital, or telephone options.

Practical Support:

Reduce travel barriers through local delivery, transport, reimbursement, accommodation, and home visits; offer loaned equipment, discounted leisure access, and food vouchers.

Cultural Competence & Support Services:

Train staff in equality/diversity, adapt dietary advice to cultural/religious needs, involve carers, and link patients to community, financial, and peer support.

What are the considerations for strategic leaders?

1. There should be a conversation to understand how a patient's prehabilitation care plan and subsequently cancer surgery will affect their life.
2. Prehabilitation services should be made available on a 'systemwide' level (i.e., across the geographical footprint of a healthcare organisation).
3. Where a prehabilitation service covers a large geographical area, activities should be spread across that area (e.g., using local healthcare facilities / community leisure centres) to minimise patient travel distances.
4. Professional communication related to prehabilitation should be shared/integrated with other professionals involved in the patient's care (e.g., primary care, surgical team).
5. Prehabilitation services should negotiate discounted access to leisure facilities, to facilitate patients' participation in exercise.
6. Prehabilitation services should loan equipment (e.g., exercise equipment, digital equipment) to patients to facilitate their engagement in prehabilitation.
7. Prehabilitation activities should be made available using digital technologies (e.g.,video-conferencing), to facilitate participation without the need for in-person attendance.
8. Prehabilitation activities should be made available on a face-to-face basis, to facilitate participation without access to digital / telephone facilities.
9. Prehabilitation services should reimburse / provide travel expenses to facilitate the attendance of patients who are less able to afford travel.
10. Patients with learning disabilities should be enabled to participate in prehabilitation, in collaboration with learning disability nurses / teams.
11. Prehabilitation activities should be made available using telephone, to facilitate participation without the need for in-person attendance.
12. Prehabilitation services should provide food vouchers to support patients who are less able to afford food to engage with dietary recommendations.
13. Prehabilitation services should provide travel / transport services, to enable attendance by patients who are less able to travel independently.
14. Where patients are unable or less able to travel to attend prehabilitation services, home visits should be offered.
15. Prehabilitation services should arrange local accommodation to facilitate the participation of patients from out of area.

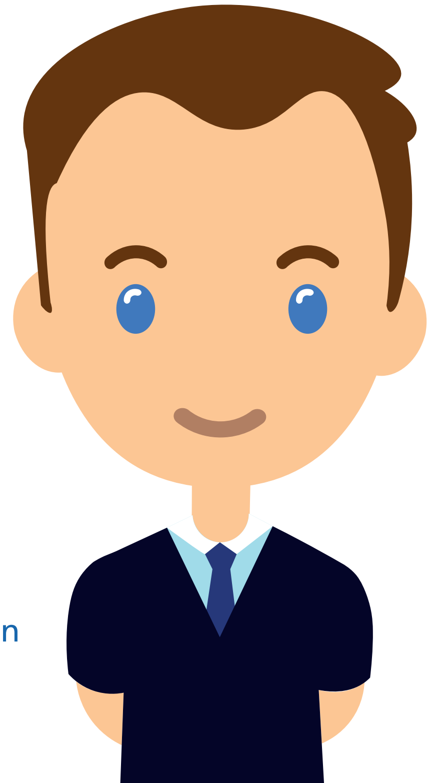

What are the considerations for operational leaders?

1. Prehabilitation services should be prepared to adapt the way they are delivered to suit the needs of individual patients, and facilitate their participation.

2. Prehabilitation services should include outreach to contact patients who do not appear to be engaging, and identify barriers to participation.

3. Interpreters should be provided for those who need them (including for sensory impairment) at each appointment.

4. The care team should be trained to improve their understanding of equality and diversity issues).

5. Prehabilitation services should provide a wide range of appointment times, to enable participation by patients who have time-bound commitments (e.g., work, caring responsibilities).

6. Prehabilitation staff who provide dietary advice should be trained on cultural and religious dietary conventions and requirements.

7. When patients are expected to undertake administration to participate in prehabilitation (e.g., booking appointments, arranging transport), they should be offered support to complete this.

8. Prehabilitation should take place in facilities which are fully accessible to patients with restricted mobility.

9. Where digital data entry is needed (e.g., during screening), patients should be offered support to complete this.

10. Digital resources (e.g., videos, images) should include subtitles / metadata.

11. Prehabilitation teams should make referrals to support services (e.g., charities, social services, social prescribers) where a need is identified.

12. Prehabilitation teams should signpost community resources (e.g., leisure centres) which have gender-specific facilities (e.g., women-only gyms).

13. Prehabilitation literature (e.g., patient information leaflets) should be made available in paper form, to facilitate engagement by patients without access to digital facilities.

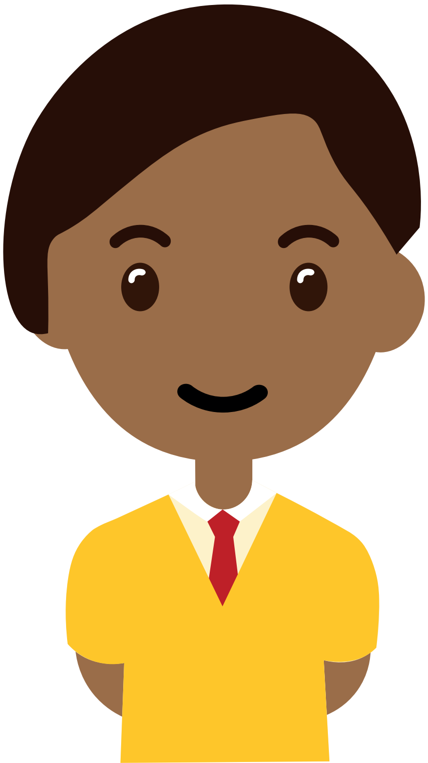

14. Prehabilitation teams should signpost patients to financial support services (e.g., charities, credit unions).

15. Prehabilitation services should refer patients who are from out of area to local prehabilitation programmes to facilitate their participation.

16. Prehabilitation services should facilitate peer support between patients.

17. Prehabilitation literature (e.g., information leaflets) should be available in languages which are commonly used by the local population.

18. Appointments to attend prehabilitation activities should be available to suit diverse religious / cultural calendars.

19. Prehabilitation services should have a clear and dedicated means of contact for patients (e.g., telephone hotline).

20. Prehabilitation services should have a group of 'key contacts' to provide advice for caring for patients with protected characteristics and vulnerable people when they are referred.

21. Prehabilitation teams should accommodate patients who wish to be cared for by people of their own gender.

22. Prehabilitation teams should signpost retailers which supply exercise clothing appropriate for patients' religious / cultural preferences.

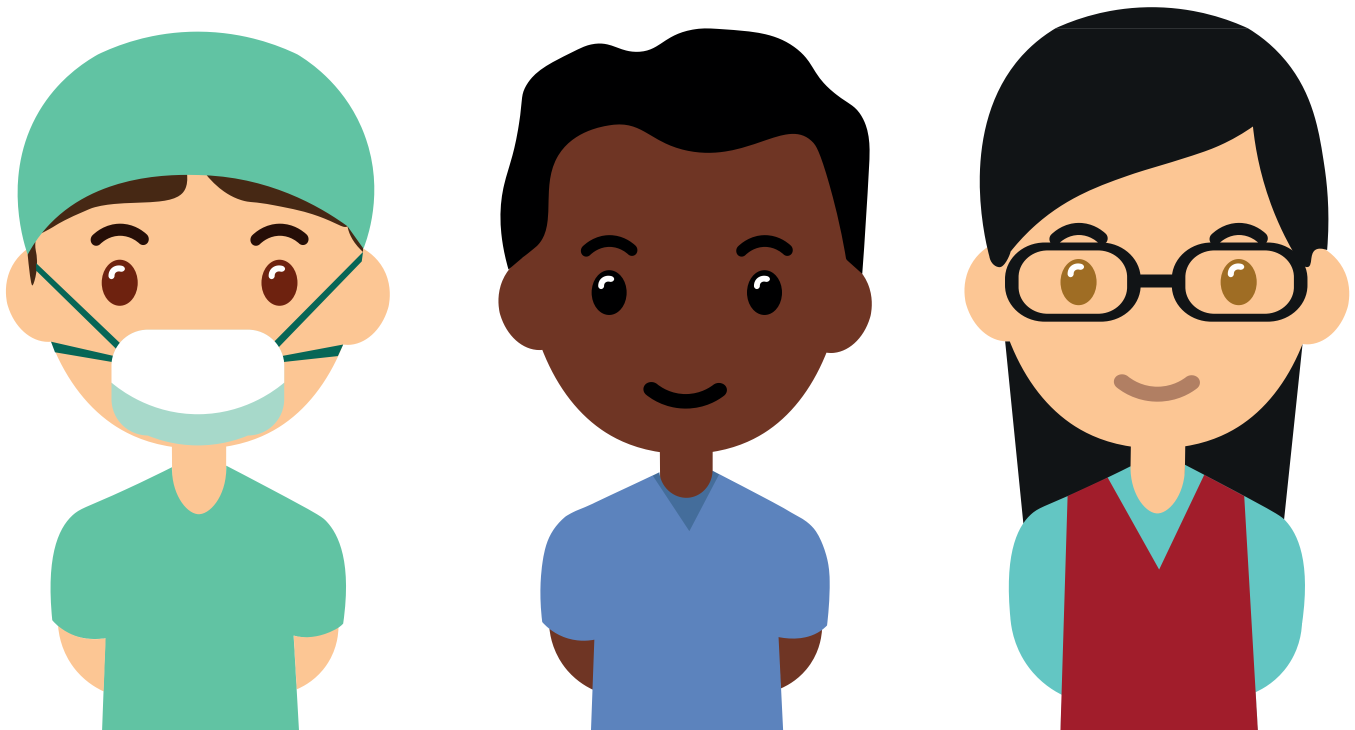

What were the considerations for practitioners?

1. Prehabilitation teams should acknowledge and normalise that patients may find the activities involved in prehabilitation challenging (e.g., due to physical fitness and / or symptoms).
2. Patients' relatives and carers should be included in prehabilitation appointments, if the patient expresses a preference for this to take place.
3. Prehabilitation teams should encourage patients to engage in physical activity based on their usual everyday activities (e.g., walking the dog).
4. Prehabilitation activities should be adapted to facilitate the participation of people living with disabilities.
5. Prehabilitation staff who provide dietary advice should accommodate patients' preferences when developing nutrition plans.

What other information is available from the PARITY study?

We have summarised our work in six academic papers (one of which has an associated podcast) and an accessible e-learning module.

The inequalities and challenges of prehabilitation before cancer surgery: a narrative review. Stewart et al, Anaesthesia, 2025. https://doi.org/10.1111/anae.16502
Podcast: https://tinyurl.com/3a7tn285

Defining criteria for quality and equity in prehabilitation services before cancer surgery: a delphi study informed by lived and professional experience. Wareing et al. European Journal of Cancer Care, 2025. https://doi.org/10.1155/ecc/9308284

Prehabilitation before cancer surgery in the UK National Health Service: what services exist, and how do they address health inequalities? Stewart et al, PLoS One, 2026. https://doi.org/10.1371/journal.pone.0336005

Addressing inequities in the design and delivery of prehabilitation in the UK: case studies of challenges, complexities and good practice. Hadley et al, Anaesthesia, 2026. https://doi.org/10.1111/anae.70286

Improving equity in prehabilitation before cancer surgery: consensus-based considerations for leaders and practitioners from a nominal group technique study. Zhang et al, Anaesthesia Reports, 2026. https://doi.org/10.1002/anr3.70085

Better by design: an innovative methodological approach to inclusive public participation for cancer prehabilitation services. Wareing et al, Forthcoming

PARITY e-learning. Forthcoming

Who was involved in the consensus workshop?

PARITY team: Cliff Shelton, Lisa Ashmore, Xiubin Zhang, Sophie Stanley, Charlotte Hadley, Laura Wareing, Ces Kulikowski.

Expert Panel: Rita Antonova, Janet Moss, Rashmi Kumar, Zoe Merchant, Tessa Renouf, Sandra Smith, Sam Moore, Clare Doney, Vicki Wester, Lesley Smith, Rachael Barlow, June Davis, Debbie Provan, James Thorp

Who was involved in writing this document?

Lead author: Xiubin Zhang
Co-authors: Cliff Shelton, Hilary Stewart, Laura Wareing, Charlotte Hadley
Advisor: John Moore
Graphic design: David Buckley

NB – some panellists asked not to be identified publicly, and we have respected that request.
